# Has Surgical Treatment Lessened the Mortality from Appendicitis?

**Published:** 1909-06

**Authors:** 


### Reviews of Books.

**Has Surgical Treatment Lessened the Mortality from Appendicitis?** By FRANK P. VALE, M.D. Pp. 165. Providence: Snow & Farnham Co. 1908.

If anyone is hunting for a good example of the way in which a writer can make a misleading use of statistics, they will find an excellent one in this book. All statistics are not necessarily misleading, but they have to be most carefully prepared to show a true correspondence. We never read more misleading ones than those contained in this book. We think the prize of 200 dollars was very badly spent in encouraging such work. Has surgical treatment lessened the mortality from appendicitis? What reasonable medical man can doubt it? Who, with any knowledge of the results obtained by surgery, could hesitate for one moment as to the answer? Suppose there were two private hospitals in the city in which Dr. Vale practices for the treatment of appendicitis, and in one no case was ever operated on, whatever the condition of the patient, and in the other operation was performed when considered necessary, which would Dr. Vale himself go to? Need we inquire? With his book of useless statistics under his arm, he would clamour for admission to the hospital where he knew, if he had a suppurative or gangrenous appendicitis, he could be operated on.

What is the value of statistics of the mortality of appendicitis which probably do not include cases of general peritonitis starting in perforation, or some other disease of the appendix ? It would take too long to criticise in detail the vast collection of statistics. Would that he had been better employed than in making them.

However, when his mind is not occupied by those useless statistics he says some very true things. For instance, on page 7 he says, " Every surgeon should be first and foremost a physician." How true is this. Whenever a surgeon undertakes the treatment of a disease, he ought to understand that disease, its physical signs and its differential diagnosis, just as much as the physician who may have had the case under his care up to that time. If he does not, he is not in a position to take the responsibility of advising surgical treatment in that particular case, and he becomes the mere human tool of the physician. Physicians sometimes consider that a surgeon need know nothing about the diagnosis of such conditions as empyema, or a dilated stomach, but that he should receive the diagnosis of the physician in all meekness and humility. But the surgeon will often find that where there is a doubt as to diagnosis, the physician is very ready to associate the surgeon with him in the uncertainty when explaining matters to the patient. The truth is, surgeons are becoming more and more operating physicians. Dr. Vale tells us of the " operating neurologist " as the latest subspeciality, and an excellent one it would be. The general surgeon, alas ! finds it impossible to be a good neurologist and general surgeon combined, and in this department of work he has no doubt to become now and then the mere human tool of the physician, though a highly-skilled one ; but even in this line of work he ought to know a good deal about the pathology and symptoms of brain and spinal cord tumours and brain abscess.

Dr. Vale says that " there must be found some point of vantage from which medicine can be viewed as a whole. If specialism develops without such means for its assimilation, one hundred years from now a corps of specialists will hover over each patient." Perhaps this will be so—if we reduce the 100 to 10—and, if so, who will inform the patient of the nature of his case, and the treatment advisable ? Will the surgeon say that operation is indicated if the physician is right in his diagnosis, and the ophthalmologist is right in his diagnosis of optic neuritis, and the aurist of suppuration in the middle ear ? And yet when a case is taken in hand by multiple specialists, who is to take the responsibility ?

There is one very interesting fact brought out by Dr. Vale's investigations which ought to discourage anyone writing another book or even journal paper on appendicitis. "Since 1896," he says, "there are indexed in the catalogue of the Surgeon-General's Library at Washington 295 monographs (including graduation theses), and 3,616 journal articles on appendicitis." There seems no fallacy here, but perhaps every thesis written by the innumerable university graduates of America is included in this collection.

